# The transition of childbirth practices among tribal women in Gujarat, India - a grounded theory approach

**DOI:** 10.1186/1472-698X-13-41

**Published:** 2013-10-03

**Authors:** Bharati Sharma, Gayatri Giri, Kyllike Christensson, Ramani KV, Eva Johansson

**Affiliations:** 1 Centre for Management of Health Services, Indian Institute of Management, Ahmedabad, India; 2 Department of Womens’ and Childrens’ Health, Unit for Reproductive Health, Karolinska Institutet, Stockholm, Sweden; 3 Common Health, A National Coalition for Maternal and Neonatal Health and Safe Abortion, India; 4 Global Health, Karolinska Institute, Solna, Sweden

**Keywords:** Childbirth practices, Medicalization, India, Grounded theory

## Abstract

**Background:**

Under the National Rural Health Mission, the current emphasis is on achieving universal institutional births through incentive schemes as part of reforms related to childbirth in India. There has been rapid progress in achieving this goal. To understand the choices made as well as practices and perceptions related to childbirth amongst tribal women in Gujarat and how these have been influenced by modernity in general and modernity brought in through maternal health policies.

**Method:**

A model depicting the transition in childbirth practices amongst tribal women was constructed using the grounded theory approach with; 8 focus groups of women, 5 in depth interviews with traditional birth attendants, women, and service providers and field notes on informal discussions and observations.

**Results:**

A transition in childbirth practices across generations was noted, i.e. a shift from home births attended by Traditional Birth Attendants (TBAs) to hospital births. The women and their families both adapted to and shaped this transition through a constant ’trade-off between desirable and essential’- the desirable being a traditional homebirth in secure surroundings and the essential being the survival of mother and baby by going to hospital. This transition was shaped by complex multiple factors: 1) Overall economic growth and access to modern medical care influencing women’s choices, 2) External context in terms of the international maternal health discourses and national policies, especially incentive schemes for promoting institutional deliveries, 3) Socialisation into medical childbirth practices, through exposure to many years of free outreach services for maternal and child health, 4) Loss of self reliance in the community as a consequence of role redefinition and deskilling of the TBAs and 5) Cultural belief that intervention is necessary during childbirth aiding easy acceptance of medical interventions.

**Conclusion:**

In resource poor settings where choices are limited and mortality is high, hospital births are perceived as increasing the choices for women, saving lives of mothers and babies, though there is a need for region specific strategies. Modern obstetric technology is utilised and given meanings based on socio-cultural conceptualisations of birth, which need to be considered while designing policies for maternal health.

## Background

Childbirth practices have been part of social, political, economic and hygienic reforms in India since the 19^th^century [1] in an attempt to combat infant and maternal mortality. From the 19^th^ century to the beginning of the 21^st^ century, reproductive reform was dominated by the ideas of overpopulation linked with general underdevelopment and poverty. The reforms for childbirth included providing a new setting for childbirth from home to hospital and a new attendant, replacing the Traditional Birth Attendant (TBA) with a qualified midwife or physician, otherwise known as a Skilled Birth Attendant (SBA) as is the current practice [2].

In spite of the emphasis on childbirth reforms, the progress in achieving institutional births and skilled attendance has been slower than desired; prior to 2006, there were 61% of homebirths in India and 45% in Gujarat, almost half of which were attended by a TBA [3].

Since the launch of the National Rural Health Mission (NRHM) in India in 2005, there has been an overwhelming emphasis on increasing institutional births by way of incentives [4]. The past five years have seen a phenomenal increase in institutional births from 39% in 2006 [3] to 73% in 2009 [5]. The emphasis on institutional births have been criticised by some grassroots organisations owing to the unpreparedness of the hospitals to receive women attracted to them through incentives [6]. These grassroots organizations see the government maternal health policies as coercive, negatively influencing the health of women.

Past research relating to childbirth has been focused on the framework of health systems, measuring compliance among women to the services offered and evaluating the services provided in order to make the health systems more robust and reduce mortality. For instance, there have been studies on the use of modern antenatal care [7,8], choice of birth place [9] and postnatal care as well as relevant determinants.

There have been a few ethnographic studies exploring childbirth practices in different parts of India [10-12]. This qualitative study complements such previous ethnographic studies adding the women’s own perspectives. Our study explores the perspectives of women on childbirth; their choices, reasoning behind decisions and conceptualisations of good/bad and normal/complicated childbirth as moulded by modernity and general developments, state policies and changing biomedical technologies. The study was carried out in the western parts of India, in a close-knit tribal society where traditional practices are more common place than among women in the mainstream rural society of India.

### International and national maternal health policies: the context

The international maternal health policies have changed from ‘Birth by trained Traditional Birth Attendants (TBA)’ , and ‘Risk screening in the antenatal period’ during the 1980’s to Skilled Birth Attendance (SBA) and Emergency Obstetric Care (EmOC) from late 1990’s onwards [13]. In congruence to this international policy, the Indian maternal health policies focused on antenatal care, risk screening and the ‘five cleans’ (clean hands, surface, blade, cord tie, and water) through the provision of safe delivery kits to trained TBAs until the late 1990’s [14].

More recently, an important international discourse influencing national maternal health policies in developing countries was ‘Every pregnancy is at risk’. As a consequence, all pregnancies should be attended by Skilled Birth Attendants (i.e. a qualified midwife or doctor) with backup being provided by health centres in case of complications. The national maternal health policy in India sees hospital births as safe births. Achieving universal coverage of births in institutions is the objective of Phase II of the Reproductive and Child Health Programme (RCH-II) [15].

Another discourse, i.e. ‘TBAs are ineffective in reducing maternal deaths’, has led to the redefinition of their role. During the decade 1987–1997, training of TBAs for safe births was part of the maternal health strategy of the Interagency Group (IAG) for Maternal Health [13]. In 1997, when reviewing the strategy progress during a meeting in Colombo, Sri Lanka, the IAG found little progress in the reduction of maternal and newborn mortality. Attention was drawn to the importance of skilled birth attendance and TBAs were not found to be skilled. The declaration of the millennium development objectives in 2000 and statement on skilled birth attendants in 2004 [2] clearly argued for a redefinition of the TBA role.

Responding to the international discourse against TBAs, the National RCH-II programme excluded TBAs as childbirth attendants and stopped TBA training, with the exception of certain districts where institutional births accounted for less than 30% of total births , p. 102 [15].

The international and national maternal health policies described briefly herein are examined later in this paper in the context of childbirth factors influencing the choices, preferences and practices of women.

## Methods

### Setting

Six districts out of total 25 in Gujarat are identified as high focus districts by the Ministry of Health, Government of India, with weak performance on health indicators including maternal and child health [16]. One such district with a predominant tribal population was selected for this study.

The tribal population in India is 8-9% compared with 14.8% in Gujarat [17] and 72% in the selected district [18]. The tribal society is described as indigenous communities, their economy and life linked to natural ecosystems and resources, dependent on agriculture, forestry and hunting. As a result of settlements invading and taking control of natural resources during the pre-colonial period and into the early post-independence period, the tribal people have lost authority over their economic resources [19,20]. There is an out-migration of tribal people to larger cities for subsistence.

The Indian population is heterogeneous in terms of religion, languages, castes, making it difficult to carry out a study on women’s childbirth perceptions and practices. The cultural homogeneity of the tribal population was one of the reasons for selecting the district in question. Unlike the Hindu population, there were no caste hierarchies. Being an indigenous community of special interest to the Government for childbirth reforms, a recent development shows women choosing to go to institutions for childbirth, with 30-40% of the women still opting for homebirths [21]. This fulfilled the objective of the study, of understanding the transition of childbirth practices from home to hospitals. The selected study district is generally less developed with an urban area of 13% compared to 48% in Gujarat, a total literacy rate of 60% compared to 81% in Gujarat and a female literacy rate of 49% compared to 71% in Gujarat [22].

The district consists of seven administrative areas or sub-districts called Blocks covering several villages, each with its own Block Health Office. Three Blocks, i.e. urban, semi-urban and rural, were selected in consultation with the District Health Office on the basis of their general development and geographic location. Table 1 describes the general development of the three blocks in terms of geographical characteristics, access by means of road, rail and other as well as the availability of modern allopathic (bio-medical or western) health facilities whether private or state-run. As seen in Table 1, the rural block was the worst off in terms of the general landscape, which was hilly and covered with forests and hence, difficult to cultivate. In addition, it was not served well by public transport and had fewer functioning healthcare facilities compared to urban and semi-urban blocks

**Table 1:** Characteristics of the selected study blocks (sub-district)

**General development characteristics**	**Urban**	**Semi-urban**	**Rural**
Physical characteristics	• Largely flat terrain	• Mix of hills & plains	• Rocky steep slopes
	• Clustered housing pattern	• Majority clustered housing pattern	• Forest cover
			• Scattered houses spread over 4 sq kms.
Accessibility (approach road, distance from town, variety of transport available)	• Good roads	• Good roads	• Good roads surrounding villages but kuccha roads inside the village
	• Situated along highway connecting other provinces	• Within 15 kms of two towns	• 30 kms. from block head quarters
	• 30 kms from district headquarters	• Connected through buses and private transport	• Buses 2 times a day
	• Connected by railways, buses, private transport		
Availability of childbirth services in Government health facilities	• Best performing round the clock government Primary Health Centre (PHC)	• Non functional round the clock PHC and CHC	• Non functional round the clock PHC
	• Non functional Community Health Centre (CHC)		• Functional subdistrict hospital 30 kms. Away
Availability of Private facilities	• Majority of district private doctors enrolled in Chiranjeevi Yojana (CY) in city near block	• Majority of private doctors enrolled in CY in city of adjoining district	• Only two CY doctors at block town

According to government norms, there is one Primary Health Centre (PHC) for every 20,000 inhabitants. Each block has one PHC providing childbirth services, day and night. Two villages were selected in the area of the PHC providing day and night childbirth services, one adjacent to and the other further away from the PHC so as to provide basis for focus group discussions and in depth interviews on the assumption that having access to a functional government health facility may influence the childbirth practices of women.

### Data collection and participants

Since the study aimed to explore childbirth perceptions, preferences and choices within a cultural context, focus group discussions with women were considered appro-priate. During the course of the fieldwork, the research team was given the chance of observing a birth both at the PHC and at home. Interviews with TBAs gave a deeper understanding of childbirth practices.

Data collection and analysis were simultaneous processes. For instance the questions during the initial focus groups explored childbirth practices in general; women’s health seeking preferences during pregnancy, childbirth and post-pregnancy as well as their preference for place of birth. The women described measures taken by them and their family in the event of pregnancy, advice sought and clinical and any other procedures carried out during each stage of pregnancy and birth.

These initial focus groups were coded and tentative categories were identified which were further explored by the next focus groups and in depth interviews. For instance the initial focus groups indicated a transition in women’s childbirth preferences across the old and new generation and also across urban, semi-urban and rural blocks which seemed to be influenced by multiple factors. This process of transition was probed in subsequent focus groups including the reasoning used by the women for choosing their preferred place of birth and their opinion on a good and poor childbirth.

The TBAs in the rural block were attending 6–7 homebirths a month although they were being discouraged by the Block Health Office as there is a thrust on 100 percent institutional deliveries by the state. TBAs were interviewed about their experiences and understanding of childbirth, their perceptions about changes in the women’s practices from past to present and experiences with the public health system.

All in all, there were eight focus group discussions, five in depth interviews, two unstructured observations and many informal discussions. Eighty-five women participated in the eight focus groups with an average participation rate of 7–11 women engaged in each discussion. However, two of the focus groups had a participation rate of 15–18 women. With the exception of two focus groups with a constant level of participation, the other focus groups saw women coming and going, often halfway through a discussion. This is one of the reasons for missing data on background characteristics for some participants (Table 2).

**Table 2:** Background characteristics of participants

**Background characteristics**	**Total %**
**Total 85 participants**	
**Age in years**	
≥20	7.7
21-30 yrs	37.2
31-40 yrs	14.1
41-50 yrs	9
≤ 50	3.8
Missing data	28
**Education**	
Illiterate	26.9
1 to 5 yrs	6.4
6 to 10 yrs	35.9
≤ 12 yrs	11.6
Missing data	19.2
**Place of last birth**	
No child	1.3
Pregnant	3.8
Home	30.8
Govt	15.4
Private	26.9
Missing data	21.8
**Number of children**	
No child	7.1
Pregnant	9.0
1 child	14.1
2 children	24.7
3 to 4 children	38.5
more than 4 children	4.7
missing data	1.3

Participating women were aged between 20 and 55 years, the majority of who were 20–35 years (Table 2) Thirty percent of the women had homebirths and 39% of them had 3 to 4 children while 5% had more than 4 children. Every woman had either a personal experience of childbirth or had been closely involved in the childbirth of a family member or neighbour.

Data was collected throughout the period of May 2011 to April 2012. The authors alternated between being moderator and note taker during the focus groups. The women were invited either through the contact of a local voluntary organisation or the Accredited Social Health Activist (ASHA) appointed by the Government under the National Rural Health Mission (NRHM). The ASHA is usually a local woman from the village appointed to mobilise and motivate the villagers to utilise the Government services on offer. The duration of each focus group was typically 1½ to 2 hours. The focus group meetings took place in common areas such as the village pre-school called the *anganwadi.* The choice of location was important as it was a neutral space and also familiar to the women seeing as antenatal clinics were held here once a month.

Field notes of informal interactions with health workers, doctors and district health managers were used in analysis. The field research team held regular meetings after each focus group and throughout the analysis process. Two meetings were organised with the larger research team to discuss the emerging findings and plan for further data collection. Notes were maintained after each meeting, which helped the analytic process.

The research team had four women and one man (KVR). The first author (BS) has a background in child development and long experience of research in the area of maternal and child health. This paper is a part of her doctoral (PhD) project investigating professionalization of midwifery in India. The second author (GG) has a background in social science and public health with experience in research and work at grassroots level, in the areas of maternal and child health care. GG had spent eight years in the district selected for the current study, working on issues of gender natural resource management and women’s health. The first and second authors have lived in the province since birth and are fluent in the local language and familiar with the local culture, which was an added advantage. The two women researchers from Sweden (KC, and EJ) are Professors at the Karolinska Institute, Sweden. Both are midwifery and nursing professionals with a doctoral education in public health and long experience of working in low and middle-income countries. Coming from the Indian Institute of Management, KVR has vast research experience in the field of public health research including maternal and child health.

### Ethics

Verbal consent was taken from the women prior to participating in focus group discussions as well as for the purpose of recording and taking notes. Since the topics of discussion were deemed sensitive, it helped that the team members responsible for collecting data were all women. The Government of Gujarat gave permissions required for the study and the Ethical Committee of the Indian Institute of Management gave the ethical clearance.

### Data analysis

**Figure 1:**
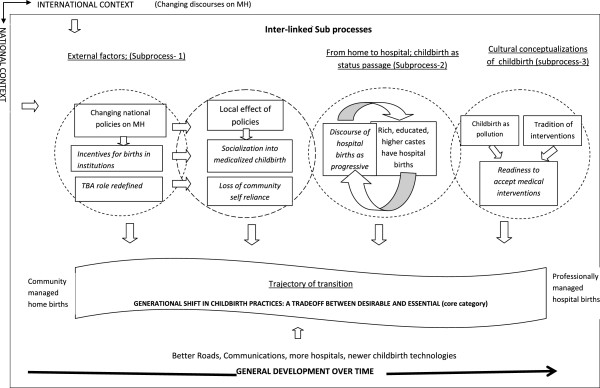
Transition of childbirth practices over generations-a model.

We used a grounded theory approach [23] to understand the central organising process of changes in childbirth practices. Grounded theory approach is an analytical approach to construct a theory grounded in empirical data using constant comparison. All focus groups and in-depth interviews were tape recorded and transcribed verbatim in the vernacular. The N Vivo software was used to assist in analysis. Because the software did not support the Gujarati language while generating queries, the first author translated the transcripts to English for further analysis halfway through the study. Considering ‘*all is data*’ [24] the data collected through focus groups, in-depth interviews and field notes, additional insights from literature on international and national maternal health policies have been used to construct the model (Figure 1) and to ‘suggest other possible meanings’ [23]. The perceptions and experiences of childbirth shared by the women have been analysed within the wider context of international and national discourses on maternal health, using the conditional matrix [23], p. 94].

**Figure 2:**
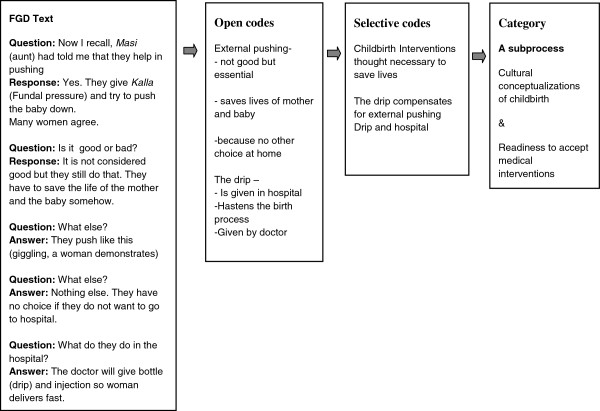
Example of the analysis moving from text to category.

The analysis process began soon after each focus group and more extensively once the transcripts were ready. The transcripts were read and an open and selective coding was performed. Codes were assigned line by line and/or to every meaning unit based on the importance of the text to the research question. Using constant comparison, codes were compared from one focus group to another and across the three blocks. During the selective coding, open codes were organised into clusters to facilitate re-reading of the material and a more focused coding. Codes were clustered to form sub-categories. The sub-categories were related and organised to represent the context, conditions and factors contributing to and the consequences leading to the transition of childbirth practices. By way of theoretical coding, this process later led to the identification of categories as sub-processes contributing to the transition of childbirth practices. An example of moving from text to category is explained in Figure 2. The process was aided by the writing of memos at each stage of the analysis, which helped in thick descriptions.

## Results

In the model (Figure 1), the phenomenon is presented juxtaposed with the larger context with the overall economic development over time (the bold arrow below the transition trajectory) and international and national maternal health context shown surrounding the model outside of the box and also as Sub-process1. The core category ‘trade-off between essential and desirable’ represents the process through which the women and their families adapted and which also shaped the transition. Three categories are interpreted as sub-processes (underlined) in Figure 1. The sub-process itself is shown enveloped in four circles: Sub-process1 titled ‘External factors’ represents the overall context and response of the national policies to the changing international discourses on maternal health and the impact of this locally. Sub-process 2 titled ‘From home to hospital; childbirth as a status passage’ and Sub-process 3 titled ‘Cultural conceptualisations of childbirth’ describe the influence of socio-cultural belief systems on childbirth practices. The circles have permeable overlapping boundaries representing interconnectedness. There were several sub-categories (italicised) explaining the consequences of these sub-processes, thus contributing to the trajectory of transition.

### The trajectory of transition of childbirth practices

The narratives of childbirth practices of women in the focus group discussions compared practices of the past and present and that of the older and younger generations. As presented in Figure 1, this change could be perceived as a trajectory, a route or movement through time and generations; a shift from home births (a community managed social event) to hospital births (a professionally managed medical event).

The women from the three selected study blocks could be placed at different points on the trajectory; the urban and semi-urban blocks to the right of the trajectory with more than 90% of childbirths taking place in institutions (Table 3) and the rural block towards the left of the trajectory with 23% of childbirths still taking place at home^a^. Nevertheless, the women were all in the process of transition either through a natural process over time or hastened by government policies as explained later in this paper.

**Table 3:** Place and distribution of births in the study district and selected blocks from April 2010-March 2011(source: District health office)

	**Urban block**		**Semi rural block**		**Rural block**		**District**	
	**Number**	**%**	**Number**	**%**	**Number**	**%**	**Number**	**%**
Total births	4949	-	6764	-	6061	-	54317	-
**Institution**	4636	94.0	6448	96	4754	77	50486	93
Public	881	19.0	803	12.8	175	3.7	9451	18.7
Private*	3755	81.0	5645	87.5	4579	96.3	41035	81.2
**Home**	313	6.3	293	4.3	1407	22.8	3831	7
TBA	312	99.6	284	96.3	1350	96	3679	96
ANM	1	0	9	3.7	45	3.1	152	3.9
Others	0	0	0	0	12	0.9	24	0.6

*Includes births in private hospitals enrolled in the Chiranjeevi Yojana and other private hospitals.

In the urban and semi-urban blocks where the majority of births occurred in hospitals, a small number of homebirths took place either because of uncomplicated labour or, or as so often was the case, because there was no time to reach a hospital as the women had waited too long for their labour to progress in order to minimise their hospitalisation time. Most women giving birth in hospital accepted medical interventions which accompany hospital births. Many of the women from the rural block favoured homebirths assisted by the traditional birth attendant and without medical interventions.

### A generational shift in childbirth practices: A trade-off between desirable and essential

The core category, ‘A trade-off between desirable and essential’, was central to the reasoning used by the women and their families in choosing between a homebirth or hospital birth and their response to the factors/conditions contributing to the trajectory of transition. The women actively adapted to these factors/conditions and made a conscious choice between the two alternatives by weighing up the advantages and disadvantages of homebirths and hospital births.

There was psychological comfort in giving birth at home, as they were managed within the community, in the presence of the birthing women’s family, friends and neighbours. Homebirths were relatively economical and convenient because the older children and cattle were not left unattended. In the rural blocks, homebirths assisted by TBAs were still the traditional way.

Hospital births were described as being alone in the labour room with strangers such as nurses, doctors or other attendants. Family and friends were not allowed. The women felt uncomfortable with the at times non-courteous behaviour of the staff. Hospital births were costlier compared to homebirths. There was fear of unnecessary interventions like episiotomies and quick caesarean sections. As a result of such possible invasive procedures, the cost of delivery and healing time after birth increased. Women in the semi-rural province mentioned the cost of C-section births ranging from USD 150–300, which is a huge amount for rural families dependent on agriculture.

Amongst the advantages of hospitals, they were described as equipped with clean linen, equipment and staff to clean up after birth. There was constant monitoring in hospitals - *‘they keep checking and giving us a probable time of birth’*. Complications were dealt with immediately, saving the lives of mothers and babies. The women were impressed by the competence of the doctors and nurses in hospitals.

With experience of home and hospital births over two generations and when weighing up the advantages and disadvantages with hospital births, the women found the advantages to outnumber the disadvantages. It was a trade-off between what they desired (comfort of home) and what they thought essential (appropriate medical attention when needed, saving lives of mothers and babies). The women had to sacrifice one thing for another.

### The childbirth context

The childbirth context is described as two categories: ‘General development over time’ , see space below the trajectory in Figure 1, and ‘External factors’ , Sub-process1,first and second circles from the left, explaining the contribution of changing national maternal health policies to the transition of childbirth practices. The trajectory of transition and constant adaptation as well as the ‘trade-off between desirable and essential’ made by the women and their families were enacted within this context.

### The local effect of national and state maternal health policies

From discussions with the Programme Managers during the visits to the district as well as with the Block Health Office, it was clear that the priority for maternal health demanded by the state headquarters (as per the current national maternal health policy) was performance on achieving institutional births, more specifically the utilization of the *Janani Suraksha Yojana* (JSY) and the *Chiranjeevi Yojana* (CY), the two priority strategies for maternal health as explained in the following section.

### Incentives for institutional births

Though aware, the women were confused about the Government’s two maternal health initiatives; The JSY, a national scheme to promote institutional births under the Reproductive and Child Health programme Phase II and the CY, a state-run initiative*.* Under the JSY scheme, the women get INR 500 for childbirth in hospital, INR 200 for the cost of transport and INR 50 for the person helping them to get to hospital [4]. Under the CY scheme, the women categorised as living below poverty line, get free delivery services in certain private maternity hospitals enrolled in the scheme, for both normal and C-section births [25]. Fourteen of the obstetricians working in the district were enrolled in the CY scheme.

According to the statistics provided by the District Health Office (not validated), out of 93% births in institutions, 81% occurred in the private sector out of which 45% occurred in CY enrolled facilities which were private facilities approved under the CY scheme (Table 3).

Women from the urban and semi-urban blocks, with a wider acceptance of hospital births, were more aware and had taken advantage of the schemes available compared to their rural counterparts who said they had never heard of the schemes.

Women from all the blocks were clearer about the Emergency Ambulance Services introduced in Gujarat in 2007 and had called the ‘108’ ambulance service number [26]. This service is also available to transfer women in labour to hospitals. The ‘108’service facility was widely used in the urban and semi-urban blocks by families and functional PHCs for referral. In case of the rural blocks the women could not use the 108 service facility very often as although the ambulance would come to the end of the road, it meant the woman having to be carried 700-800 metres up and down the hills to reach the ambulance.

These initiatives, especially the emergency transport, has hastened the trend towards hospital births in the urban and semi-urban blocks by removing two important barriers that prevent women from going to hospitals, that is, the cost of transport and doctor’s fee.

### Redefinition of the role of TBA

With increased access to hospitals for childbirth, the women in the urban and semi-urban blocks said that there were very few active TBAs practicing in their villages. The current role of the TBA is not that of a birth attendant but a partner with the government health facility services to promote modern childbirth practices and motivate women for antenatal care, provide support to women during hospital births and facilitate modern practices of newborn and maternal postnatal care. Because the average TBA only attends a couple of births, she has lost or is in danger of losing her skills and the community is loosing its self-reliance in childbirth. Shown as part of Sub-process1 in Figure 1, the redefinition of the TBA role has contributed to the shift towards the hospital and vice-versa. This is effectively what the women in the urban province said:

Response 1: Earlier there were no such facilities (hospitals). Now, no one is available to attend delivery at home. Earlier, elder people knew everything (how to deliver). Now people do not know.

Response 2: Also 108 (ambulance service) is available so there is no cost for transportation.

Response 3: Yes, what we mean is that we do not get skilled persons. So, in that situation (when people had the skill) they used to have home delivery. No one went to the hospital before.

The majority of homebirths in the rural block were attended by TBAs, which was both a necessity as she was the only choice available and preferred because she was known and trusted. The TBAs took the risk of assisting complicated births such as breech presentations at home. Under pressure from the Government to reduce maternal deaths, the Block Health Office is steadily controlling the TBAs by giving them incentives to bring birthing women to the hospitals, that are four times more rewarding than the fees paid by the families to attend a birth. As described by one of the TBAs:

Q: Last time (8 months ago), when we came to see Kamla’s (name changed) home delivery, her mother-in-law asked me not to take her to the hospital

A: Yes because you had arrived by car…

Q: Yes. She was scared of the hospital. Now, from what you have said, it seems the majority of women go to hospitals?

A: That is because we do not get anything for home deliveries. What can a poor family pay me? INR 25 or some corn at the most. If you take the woman to the hospital you get INR 100 per delivery.

The supply of safe delivery kits to the TBAs for added hygiene during home births has been discontinued. TBAs are registered and called to regular meetings by the block health office where they are given clear and repeated instructions to bring birthing women to hospitals. Thus the process of deskilling the TBAs has begun in this block. The natural pace of change from homebirths to hospital births is being ‘fast tracked’ or hastened, imposing hospital births on women through incentives and disincentives in an effort to reduce maternal and child mortality.

The TBAs in this block described how the younger generation does not want to learn from and take on the professional skills of existing TBAs because of this pressure to submit women to hospital.

### ***Socialisation into medicalised childbirth***

The process of modernising childbirth practices that has been part of the primary health care programme of the Government of India since post independence takes many forms with emphasis currently being placed on hospital births. As a result and as shown in the second circle of Figure 1, there is a ‘Socialisation into medicalised childbirth’. The definition of socialisation here is ‘the process of adapting to a social group’ , which in this case constitutes the entire health system channelled through doctors, nurses and midwives.

This socialisation happened with experiences of the self and peers within the medical system. The Indian Government’s Maternal Health Programme has been offering free maternal health services since the time of independence in the form of antenatal and postnatal checkups in the village, health education by way of door to door contacts and child immunisation through its outreach programme. The acceptance of medical childbirth practices is evident from the following response given by women in the urban block:

If one month and 10 days are gone we take her to the hospital to get a urine test.........the urine test will tell us whether she is pregnant or has just missed her period. Then the treatment begins. Then they call us every month and then once in 15 days or so we bring the woman for a checkup....to check her weight, and blood pressure.......They ask the woman to lie down and check her abdomen for the growth of the child…..

Repeated contacts with health workers have served as points of influence where the health workers, representing medicalised childbirth practices, counsel mothers to choose hospital births.

In the urban and semi-urban blocks, where hospital births are accepted to a greater extent (Table 2), one block had a functional primary health centre and a permanent and a trusted Auxiliary Nurse Midwife (ANM) with 15 years of experience in childbirths (Table 1). The other block had easy access to private hospitals run by obstetricians and the means to afford private services. Both blocks provided regular outreach services and thus, regular antenatal care programmes aiding the socialisation into medical childbirth practices. This socialization got further embedded when there was evidence that women who had hospital births were benefiting from the successful handling of life threatening complications.

At present, the incentive schemes and the free transport seems to contribute further to the process of socialisation as seen in the following excerpt from a focus group discussion in the semi-urban block:

Q. Did the women start to go to the hospital because of the Chiranjeevi Scheme or did they go anyway?

A. Because of Chiranjeevi (scheme). We get money.

Q. OK. What is your opinion on the scheme?

A. We consider it good.

All women: Yes it is good. ....We get good treatment. They give money as well as treatment to both mother and baby.

The women in the rural block had limited exposure to medicalised childbirth practices as the government facilities were a long way away and non-functional, the antenatal care irregular and limited and there were fewer private facilities. In spite of the free transport and hospital services offered under the *Chiranjeevi Yojana*, the women could not, because of extreme poverty, afford the miscellaneous expense of going to the hospital. Consequently, the women in the rural block had less opportunity of getting socialised into medical childbirth practices.

### From home to hospital; childbirth as a status passage

The category/Sub-process 2 ‘From home to hospital; childbirth as a status passage’ , shown as the fourth circle in Figure 1, borrows the concept of status passage from Glaser [27] p. 1–4. Status passages occur in societies and refer to a journey or mobility from one social status to another that may be desired or undesired by the passagee and may be either individual or collective. This sub-process/category shows childbirth as a terrain and part of the means needed to achieve a higher social mobility and change in social status.

### Discourse of hospital births as progressive

The sub-category ‘discourse of hospital births as progressive’ captures the various elements that contribute to a status passage. Hospital births are viewed as part of a general socio-economic progress, as a normal course of development just like changing from a bullock cart to a motor vehicle for transport. *‘With changing times comes a changing society and new practices’*- Childbirth practices have in a way changed with the changing patterns of today’s consumer markets.

Hospital births are also seen as intergenerational differences similar to the evolving differences in the fashion styles favoured by older and younger generations. *‘The younger generation lives for the present moment while the older generation lives for yester years…’* Hospital births are attributed to changing lifestyles. With mechanised household equipment such as the electric grinder, interior water taps, etc., women have less physical labour and hence, weaker muscles and lower levels of physical fitness. So, being weaker compared to the women of earlier generations, the modern woman is more likely to require a hospital birth.

The place of birth is related to social class and complex imagery of modernity and a rise in social status. As expressed by one TBA, the ‘*Sudhrela’* or ‘the improved ones’- i.e. educated rich women - have hospital births, reinforcing the discourse shown as a loop in Figure 1. Moreover, the rich women went to private hospitals while the poor and lower caste women went to the state-run hospitals. Using private facilities for childbirth, despite being more expensive, was seen as more progressive. The discourse ‘hospital births as progressive’ implies that if relinquished, the preferences of women normally shift from home to hospitals as part of the general progress and intergenerational change.

### Cultural conceptualisations of childbirth

Because parturition is considered a time of impurity, homebirths had to take place outside either in the cattle shed or a room outside the main home, on a cot woven with jute without any mattress, so that the cot could be washed and reused. The TBA or a relative attending the birth would clean up after the birth though this was considered a defiling task. Even if the tribes do not have a caste hierarchy like the Hindus, the TBAs still faced discrimination as they handled childbirth impurities, one of the reasons why the professional practice of TBAs is not expanding. While describing hospital births or discussing advantages of hospitals, the women invariably mentioned, *‘we do not have to do anything, there is someone to clean’ *, indicating that they were happy to shift the impurities from home to hospital.

The description of homebirth practices by women included interventions introduced during labour to shorten the duration of labour described by the sub-category ‘long tradition of interventions’ in Figure 1. The women believed that the birthing woman needed someone else to assist actively in pushing externally to get the baby out. Applying external pressure is called ‘*Kalla*’. A gentle *Kalla* was given when the head of the baby was visible and a heavy *Kalla* when there was delay in birth or after the birth if the placenta was not expelled. This was practiced by nurses and doctors also in government and private hospitals also. In spite of being educated in western medical childbirth practices, ‘*kalla*’ is deemed a necessity among medical professionals.

The women equated the duration of labour with the pain intensity. So they felt that ‘increasing the pain’ would lead to a hastier birth. The women from the urban and semi-urban provinces saw ‘the drip to increase the pain’ or “injections to increase the pain’ as part of a good childbirth as seen in the following excerpt from a village in the urban block:

Q: Do problems such as…… uterus coming out or leaking urine …happen here?

Respondent 1: This used to happen when the practice was to push hard (external fundal pressure). Now they do not push anymore.

Respondent 8: Now they just do it with the bottle (drip)….. in the hospital they give the drip and injection so the delivery happens normally.

Respondent 4: Once the head appears, they just give a gentle push (externally)

Giving drip and injections to augment the labour as practiced by the hospitals seem to have replaced the use of *Kalla* or its importance in the minds of the women. The belief that it is necessary to intervene during labour either by using *Kalla* or increasing the pain meant that there was a ‘readiness to accept medical interventions’ , which has also aided the acceptance of medicalised childbirths.

Invasive interventions such as episiotomy and C-sections were still unacceptable and part of the description of a ‘bad’ childbirth. Higher costs, a longer recovery period and a general fear of surgery were factors associated with such invasive interventions.

On the other hand, the women in the rural block, having a minimal exposure to medical childbirths, considered a good delivery to be ‘one that happens on its own’ although *Kalla* was practiced here too.

## Discussion

A myriad of complex factors have facilitated the transition of childbirth practices from home to hospitals amongst the tribal women of Gujarat influencing their childbirth choices, preferences and practices. The model (Figure 1) explains the transition and its contributing factors in the context of changing childbirth markets and general overall development, newer obstetric technologies and international and national childbirth policies promoting western medical hospital births.

Though the use of focus groups was considered the most appropriate method for listening to women’s voices and understanding their perspectives of childbirth in their cultural environment, there were certain limitations. The research team found it difficult to restrict number of women as many came out of curiosity and it would have been impolite to refuse their participation. It was also difficult to get the women to stay against their wishes as many had left their young children at home, or had to go out to work. Since participation was not constant from the beginning to the end, it was not always possible to follow the recommended semicircle sitting arrangement and maintain the identity of the respondents [28].

We were unable to separate the groups of younger and older women, as younger women are usually not permitted to participate in any activities involving outsiders without being accompanied by an elder relative or friend. It is likely that their responses especially those of the younger women got restricted. Most of the older women were either mother-in-laws or elder sister-in-laws of the younger women. We included an experienced moderator (the second author) in the team who could sense if there was some hesitation amongst the women and deal with it. We spent some extra time in explaining our purpose of the study and informing them about their right to refuse to participate. We focussed on individual experiences of women which they were eager to share and also put them at ease. Questions seeking their opinions were woven in in-between. Hence we felt that all women participated actively. In-depth interviews and field observations also helped in triangulation of the FGD narratives during analysis.

### Interpretations of medicalisation of childbirth

The medicalisation of childbirth is a global phenomenon viewed from different perspectives. Within mainstream discourses, a medicalised childbirth is viewed as a sign of economic, social and medical progress and an indicator of safe childbirth practices [29]. However, advanced technologies, monitored pregnancies and specialist intervention have also been subject to criticism. The medicalisation of childbirth has been criticised as a violation of the autonomy of women by feminist critics [30,31]. Sociologists compared childbirth in industrialized and unindustrialized societies and found contrasting negative aspects of medical hospital births and positive aspects of traditional homebirths [32]. Medical anthropologists associate medicalisation of childbirth with dehumanisation of birth [33]. The literature related to the underpinning ideology and philosophy of midwifery focuses on the autonomy of birthing women [34] and natural childbirth opposed to medical birth in addition to the discourse against medicalization of childbirth. With births becoming more dependent on technology, midwives perceive their role as either diminishing or in need of redefinition [35] in order to *give back the birthing power to women*[36,37]. In some countries such as Australia [38] and Ireland [39], women and midwives appeal for supportive policies for homebirths.

In India the poor women have limited choices, health centres and health providers are inequitably distributed [40], health systems are weak and the maternal and neonatal mortality rates are high. The homes of poor women lack basic facilities such as running tap water round the clock making it difficult to maintain hygiene during labour. The discourses against medicalization of childbirth in the western countries do not seem to be fully relevant for these women. As seen in this study, improved access to hospitals has increased the choices available to women. The women and their families welcome medical attention during childbirth, as lives are saved. However, this acceptance is partial as the women are against what they see as at times avoidable interventions such as C-sections and routine episiotomies.

### Growth of health sector, maternal health policies and medicalisation of childbirth

The general economic development has lead to growth in the health infrastructure. In India, the number of allopathic hospitals increased almost 300 fold from the 1970s to 1990s [41]. The growth seen in the health sector has been taken over by the private sector. Wherever there is higher economic growth, the private sector -usually restricted to urban areas - also spread to rural areas [41] such as Gujarat. One of the reason for this growth in the private sector is the underfunding and underperformance of the public sector [42]. Gujarat has more than 2,000 obstetricians registered in the Gujarat section of the Federation of Obstetricians and Gynaecological Societies of India. With easy access to specialized obstetric care come new obstetric technologies such as ultrasound, fetal heart monitors and interventions like drugs to augment labour, episiotomies and Caesarean sections, which have penetrated to rural areas of Gujarat.

The emphases put on SBA and EmOC by the international maternal health strategy are interpreted as institutional births by the national maternal health policies, equating institutional births as safe births. The JSY and CY represent the interpretation of the Government that hospital births are safe births and the outcome of the underperformance of public health sector and the growth of the private health sector. In spite of the criticism [6], these policies have contributed to the transition of childbirth practices. The transition towards hospitals births found in the current study, was influenced by the inequitable distribution of health centres and providers [40]. For instance the rural block in the current study where majority of the births were still at home, had only two obstetricians enrolled through CY and none present in the government hospital during the period of data collection.

It is difficult to say whether the deskilling of the TBA has happened because of the emergence of medicalised hospital births or whether women have had no choice but to go to hospitals for births because of the TBA being deskilled. Either way, the role redefinition of the TBA has led to the loss of community self-reliance in childbirth and indigenous systems of knowledge [43]. Wherever there has been general economic development and access to modern medical facilities, hospitals have provided an alternative to women and contributed to the transition from home to hospitals. However, when the transition is hastened in the absence of general economic development and functional medical facilities, the deskilling of the TBA could take away the only choice available to women as seen in the case of the rural block in this study.

In spite of the many movements by voluntary organisations to integrate the knowledge of the TBAs into modern childbirth practices in order to make them safe and culturally appropriate birthing options for women, the health administration is making sure that the TBAs do not attend births as seen in this study. For example, the Maitrika Project [44] and the TBA Association in Gujarat, mobilised by non-governmental organisations (NGOs), have specifically asked for the TBAs to be retained for underserved areas. The Jeeva Project challenges top-down TBA training and the exclusion of TBAs and their healing modalities from the health services system [45].

### Hospital births as a status passage

The discourse of ‘hospital births as progressive’ found in this study is congruent with the argument that reproduction also provides an environment for people to conceive new cultural futures and re-organise and reconceptualise their world [46].

A study in south India exploring the impact of modernity on conceptualisations and practices of childbirth [11] concluded that bio-medical childbirth was accepted by women and viewed as ‘a culture in the making’. Another study investigating childbearing and kinship in middle class women in the east of India, also concluded that middle class women perceived state-of-the-art medical treatment as part of their rights and privileges [12], just as hospital births was seen as a status passage in this study.

### Cultural conceptualisations of childbirth and interpretations of medical interventions

Modern obstetric technologies are interpreted and consumed differently depending on the social conceptualisations of childbirth that exist in a culture. For example, the women in this study associated the intensity of their labour pains with the speed of progress in labour requesting labour augmentation in order to increase the pain. Receiving oxytocin injections was part of their perceptions of a ‘good’ delivery. The capacity to bear pain or the pain threshold reflects the women’s reproductive power [11]. As expressed by the women in this study, the women of the older generations did hard physical labour, which increased their physical strength as well as their pain threshold.

A study in rural Rajasthan [47], a province adjoining Gujarat,involving the observation of 2,301 births found that external fundal pressure was applied 94% of the time at home and a little lesser in health institutions depending on the size of the health centre. Oxytocin was given 39% of the time at home if modern care providers were present during homebirths and 93-97% in health institutions. Similar unmonitored use of oxytocin, even during home births, was found in Uttar Pradesh [48,49] and in Tamilnadu [11].The studies also found that women requested the drip to increase labour pains.

Oxytocin has been freely available as part of the skilled birth attendant policy of the Government of India [50]. The Auxiliary Nurse Midwife also known as the Female Health Worker at peripheral institutions were given permission and training to inject oxytocin as part of managing the third stage of labour to prevent postpartum haemorrhage (PPH), one of the leading causes of maternal deaths in India.

However as described in this and other studies oxytocin is used during the 2^nd^ stage of labour also to shorten the duration of labour. This may not really reduce maternal deaths. High dosages create hyper stimulation, which can lead to precipitate labour, perineal tears, uterine rupture and fetal distress [51].

### Bringing together the desirable and the essential

The trade-off between desirable and essential elements of childbirth, which guide the women in their choice of birthplace, implies that although the women have shifted to hospitals for birth they are not fully satisfied with the quality of services provided, especially from a psychosocial perspective. In countries where midwives are autonomous and empowered, they have helped to preserve the ‘women-centeredness’ of childbirth services. The World Health Organization recommends “safe care close to the women” emphasizing community based midwife led care as most efficient for ensuring good quality and culturally sensitive maternal and newborn health services [52].

As early as 1946, the Bhore Committee report had suggested specialized education and a separate cadre of midwives [53] for more efficiency in training and retention of professional interest and skills of midwives in India. Even today midwifery is integrated into nursing in India not having professional midwives practicing midwifery to its fullest extent [54].

Investing in midwives would be a way to bring the desirable and essential together for India at both the community level and in hospitals, as illustrated by a Cochrane review of 11 clinical trials involving 12, 276 women randomly assigned to midwifery led care and that of medical professionals (specialists /family physicians). The review concluded that midwifery led care was in many ways beneficial to mothers and babies with no identified adverse effects [55]; a reduction in regional analgesia with fewer episiotomies and instrumental births, increased chances of woman being cared for by a midwife that she knows, women feeling in control during labour, having a spontaneous vaginal birth and initiating breast feeding. Midwives were more likely to favour and practice aspects of social and psychological support [56]. Therefore the way forward for India would be to recall and fulfill the promise of the Bhore committee and invest in producing fully qualified midwives.

## Conclusions

In resource poor settings where choices are limited and where mortality is high, women easily accept hospital births as a better option to save lives. Maternal health policies and strategies have been an important contributor towards the transition from home to hospital in regions with a good general economic development.

However, in difficult regions with poor economic progress and where it is not possible to ensure hospital births, the same strategies may not work. Instead of taking away the limited existing choices available to the women, in terms of homebirths by TBAs there is a need to understand, respect and integrate cultural interpretations of childbirth with the maternal health policies. The health system needs to find innovative and effective ways to strengthen midwifery and ensure the availability of and accessibility to midwives at community level.

Furthermore, this study finds that modern obstetric technology is interpreted, utilised and given meanings on the basis of socio-cultural conceptualisations of childbirth. These cultural interpretations should be considered in programme and policy designs for organising maternal health services. There is a need to pilot test strategies and create local evidence for policies prior to a wider implementation.

## Endnotes

^a^Although these statistics are not validated, there is no other source of information. The women in the urban and semi-urban blocks did seem to have stronger preference for hospital births compared to the rural block. The district level household survey 2007–08 puts the figure of institutional births for the entire district as 60.1% (DLHS-III, 2007–08), for which the data was collected between 2004–06.

## Abbreviations

ASHA: Accredited social health activist; ANM: Auxiliary nurse midwife; CHC: Community health centre; CY: Chiranjeevi yojana; DLHS: District level household survey; EmOC: Emergency obstetric care; IAG: Interagency group; INR: Indian rupee; JSY: Janani suraksha yojana; NRHM: National rural health mission; NGO: Non government organization; PHC: Primary health centre; RCH: Reproductive and child health; SBA: Skilled birth attendant; TBA: Traditional birth attendant.

## Competing interests

The authors declare that they have no competing interests.

## Authors’ contribution

BS drafted the manuscript and all authors contributed to subsequent drafts and revisions of the paper. KVR, BS and GG developed the study protocols with inputs from KC and EJ. BS and GG prepared tools for data collection and carried out the focus groups and in depth interviews. They also did the initial coding and were joined by KC and EJ for further analysis. The authors have read and approved the final manuscript.

## Pre-publication history

The pre-publication history for this paper can be accessed here:

http://www.biomedcentral.com/1472-698X/13/41/prepub
